# A critical review of the methodology of ovine lumbar interbody fusion studies and recommendations for future study design

**DOI:** 10.1302/2046-3758.151.BJR-2025-0300.R2

**Published:** 2026-01-21

**Authors:** John von Benecke, Zoya Khan, Jane McLaren, Kevin Shakesheff, Nick Birch

**Affiliations:** 1 Locate Bio Ltd, Nottingham, UK; 2 University of Nottingham, Nottingham, UK; 3 Keele University, Keele, UK; 4 Bragborough Health and Wellness Centre, Daventry, UK; 5 Orthoson Ltd, Oxford, UK

**Keywords:** Spine, Ovine, Interbody, lumbar interbody fusion, Sheep, animal models, intervertebral discs, radiography, vertebrae, autograft, spinal fusions, disc height

## Abstract

**Aims:**

Interbody fusion stabilizes the spine by promoting bony growth between vertebrae. Large animal models have physiological and biomechanical similarities to human spines that can provide safety and efficacy data before human use. Sheep models are well validated and are the model of choice to improve understanding of fusion processes by allowing post-mortem analyses of tissues unavailable in human studies. They should be consistently designed to allow appropriate translation of the results into clinical practice. This paper investigates the methodological rigour of ovine lumbar interbody fusion (LIF) studies, and proposes recommendations for researchers designing future studies.

**Methods:**

PubMed and the Cochrane library were searched up to 20 December 2024. The search terms were 1) lumbar AND ("in vivo" OR "animal model") AND "fusion" AND (interbody OR cage OR anterior) and 2) (lumbar AND ("in vivo" OR "animal model") AND (spine OR intervertebral disc) AND (sheep OR ovine) AND fusion).

**Results:**

A total of 323 papers were identified; 48 studies were included after rejection of non-spinal references, duplicates, and non-English-language papers. Data regarding 993 animals and 1,668 fusion levels were examined. Animal ages varied from six months to nine years. Cages were used in 88% of studies, with a wide range of sizes. The commonest assessments included radiography, histology, and mechanical testing. The in-life phase of the studies varied from one week to three years. High risk of bias was evident in all papers, especially considering 1) treatment allocation sequences, 2) housing randomization, 3) blinding of caregivers, and 4) outcome assessment randomization.

**Conclusion:**

This review demonstrates variability in the design of ovine LIF studies, highlights features likely to limit the translatability of results into clinical practice (sheep age and interbody cage size), shows high risk of bias in the published literature, and makes recommendations for future studies.

Cite this article: *Bone Joint Res* 2026;15(1):58–72.

## Article focus

- Critical review of the methodological rigour in ovine lumbar interbody fusion (LIF) models (1996 to 2024), synthesizing 48 studies covering 993 sheep and 1,668 levels to characterize design variability and risk of bias.
- Highlights two major controllable determinants of clinical translatability: 1) skeletal maturity of the sheep and 2) interbody cage size relative to native disc height/endplates.
- Proposes a pragmatic ‘Nottingham Utility Score’ (NUS) as a framework to assist in the evaluation of ovine LIF studies for translatability to clinical practice.

## Key messages

- The ovine LIF literature is highly heterogeneous, with generally high SYRCLE risk-of-bias scores (mean 6.5 (SD 1.6)), making comparison between studies of limited value.
- The common use of immature animals and oversized interbody cages is highly likely to overstate apparent healing endpoints in those studies.
- Future studies should pay increased attention to the ARRIVE guidelines, SYRCLE’s risk of bias, and translatability measures such as the NUS.

## Strengths and limitations

- This review identifies two key limitations in the translatability of many LIF papers, and introduces a weighted NUS framework to help readers rapidly benchmark study utility.
- Its conclusions are constrained by the heterogeneity of the LIF papers analyzed, and the non-English-language exclusion.

## Introduction

Spinal fusion is a widely used procedure for treating certain degenerative spinal conditions. In a large USA survey, elective lumbar fusion volumes nearly tripled from 1,227 in 2011 to 3,330 in 2019, with a shift toward interbody fusions and a decline in posterolateral fusions.^1^ This reflects evolving indications, techniques, and implant availability, driven by advances in clinical research.^2-5^ Despite these changes, reoperation rates remain high.^6^ Symptomatic fusion failure is a leading cause of reoperation, highlighting the need for better patient selection,^7^ improved implants,^8^ and more efficient bone graft materials.^9,10^

In vivo preclinical testing is critical for developing and validating surgical innovations.^11^ Large animal models play a central role, offering greater anatomical and physiological relevance than small animals, and helping bridge the gap between the bench and the bedside. However, meaningful results require models that closely simulate the clinical scenario, with appropriate species, conditions, and outcome measures.^11^

Accounting for differences in posture (biped vs quadruped), loading, and bone healing is essential in orthopaedic research. Sheep are among the most relevant large animal models for spinal fusion, due to similarities in spinal anatomy, biomechanics, and bone structure to humans.^12-16^

This review evaluates the methodological rigour of ovine lumbar spinal fusion studies, identifying key design issues and limitations. It offers evidence-based recommendations to enhance the translational value and comparability of future studies, thereby improving their relevance to clinical practice.

## Methods

### Search strategy

To identify relevant studies, we developed three search terms as set out in Table I which were used to query the PubMed and Cochrane databases. These searches were performed on 20 December 2024, and included results up to that date.

**Table I.** Search terms applied to the methodological review.

Search line	Search 1	Search 2	Search 3
1	Lumbar AND	Lumbar AND	Lumbar AND
2	(“in vivo" OR "animal model") AND	(“in vivo" OR "animal model") AND	Interbody AND
3	fusion AND	(spine OR intervertebral disc) AND	(ovine OR sheep)
4	(interbody OR cage OR anterior)	(sheep OR ovine) AND	
5		fusion	

### Study inclusion and exclusion criteria and selection process

Duplicate results were identified using Rayyan (USA) and were removed. Subsequently, the titles, abstracts, and key words were assessed using the inclusion and exclusion criteria shown in Table II.

**Table II.** Inclusion and exclusion criteria applied to the results of the search.

Inclusion criteria	Exclusion criteria
Ovine lumbar interbody fusion studies English-language publications	In vitro or ex vivo studies Human clinical or cadaver studies Animals other than sheep Not lumbar interbody fusion studies Not primary research Discussion of results published elsewhere

**Fig. 1:**
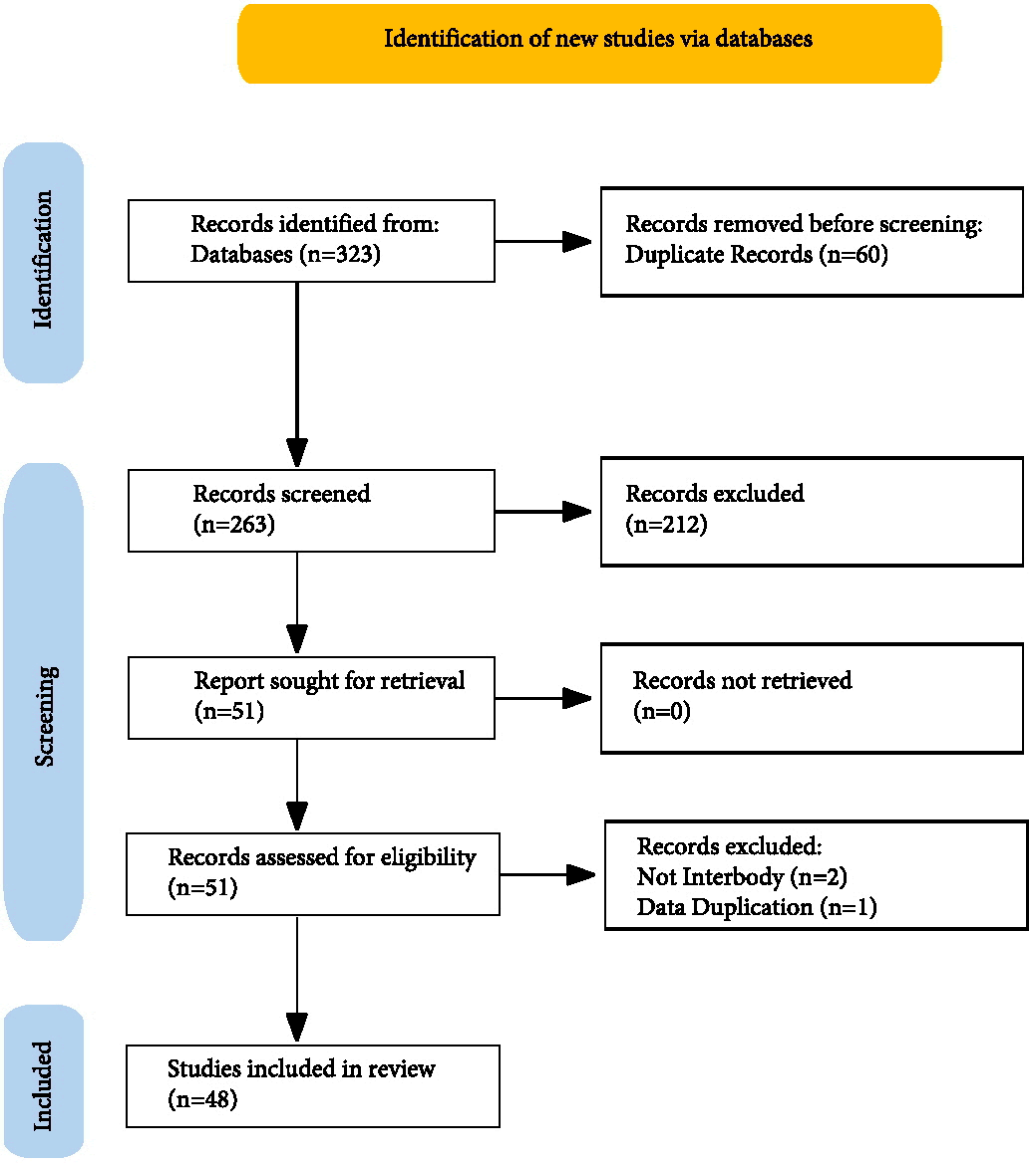
PRISMA diagram showing the process that resulted in the 48 included studies.

As shown in Figure 1 below, after review of the full text of the selected papers, a further three were excluded for the following reasons: two papers were not interbody fusion studies, and one paper appeared to include duplication of data contained in a later paper.

### Risk of bias analysis

The SYRCLE Risk of Bias (ROB) tool for Animal Studies was used to assess potential bias in the included studies.^17^ Specifically designed to address limitations of the Cochrane ROB tool when applied to animal research, SYRCLE includes ten items covering common biases (Table III). Each item was scored “yes” (low risk) or “no” (high risk), as recommended. The tool’s designers advise against summing scores for individual papers due to varying item weightings, therefore this review assigned values of 0 (“yes”) and 1 (“no”) to allow aggregate analysis across all 48 studies.

**Table III.** SYRCLE’s tool for assessing risk of bias.

Item	Type of bias	Domain	Description of domain	Review author’s judgement
1	Selection bias	Sequence generation	Describe the methods used, if any, to generate the allocation sequence in sufficient detail to allow an assessment whether it should produce comparable groups.	Was the allocation sequence adequately generated and applied?
2	Selection bias	Baseline characteristics	Describe all the possible prognostic factors or animal characteristics, if any, that are compared in order to judge whether or not intervention and control groups were similar at the start of the experiment.	Were the groups similar at baseline or were they adjusted for confounders in the analysis?
3	Selection bias	Allocation concealment	Describe the method used to conceal the allocation sequence in sufficient detail to determine whether intervention allocations could have been foreseen before or during enrolment.	Was the allocation adequately concealed?
4	Performance bias	Random housing	Describe all measures used, if any, to house the animals randomly within the animal room.	Were the animals randomly housed during the experiment?
5	Performance bias	Blinding	Describe all measures used, if any, to blind trial caregivers and researchers from knowing which intervention each animal received. Provide any information relating to whether the intended blinding was effective.	Were the caregivers and/or investigators blinded from knowledge which intervention each animal received during the experiment?
6	Detection bias	Random outcome assessment	Describe whether or not animals were selected at random for outcome assessment, and which methods to select the animals, if any, were used.	Were animals selected at random for outcome assessment?
7	Detection bias	Blinding	Describe all measures used, if any, to blind outcome assessors from knowing which intervention each animal received. Provide any information relating to whether the intended blinding was effective.	Was the outcome assessor blinded?
8	Attrition bias	Incomplete outcome data	Describe the completeness of outcome data for each main outcome, including attrition and exclusions from the analysis. State whether attrition and exclusions were reported, the numbers in each intervention group (compared with total randomized animals), reasons for attrition or exclusions, and any re-inclusions in analyses for the review.	Were incomplete outcome data adequately addressed?
9	Reporting bias	Selective outcome reporting	State how selective outcome reporting was examined and what was found.	Are reports of the study free of selective outcome reporting?
10	Other	Other sources of bias	State any important concerns about bias not covered by other domains in the tool.	Was the study apparently free of other problems that could result in high risk of bias?

## Results

### Studies evaluated

A total of 48 studies, published between 1996 and 2024, were evaluated. The citations are shown in Table IV.

**Table IV.** Citations of the 48 papers included in the review.

Year of publication	First author	Citation
1996	Sandhu	Distractive Properties of a Threaded Interbody Fusion Device. *Spine (Phila Pa 1976)* 1996;21(10):1201-1210
2000	Steffen	Posterolateral and Anterior Interbody Spinal Fusion Models in the Sheep. *Clin Orthop Relat Res.* 2000;371:28-37
2000	Toth	Direct Current Electrical Stimulation Increases the Fusion Rate of Spinal Fusion Cages. *Spine (Phila Pa 1976)* 2000;25(20):2580-2587
2001	Hadjipavlou	Plaster of Paris as bone substitute in spinal surgery. *Eur Spine J*. 2001;10:S189-S196
2001	Magin	Improved Lumbar Vertebral Interbody Fusion Using rhOP-1 A Comparison of Autogenous Bone Graft, Bovine Hydroxylapatite (Bio-Oss), and BMP-7 (rhOP-1) in Sheep. *Spine (Phila Pa 1976)* 2001;26(5):469-478
2001	Steffen	Porous tricalcium phosphate and transforming growth factor used for anterior spine surgery. *Eur Spine J*. 2001;10:S132-S140
2002	Blattert	Successful Transpedicular Lumbar Interbody Fusion by Means of a Composite of Osteogenic Protein-1 (rhBMP-7) and Hydroxyapatite Carrier. A Comparison With Autograft and Hydroxyapatite in the Sheep Spine. *Spine (Phila Pa 1976)* 2000;27(23):2697-2705
2002	Sandhu	Histological Evaluation of the Efficacy of rhBMP-2 Compared With Autograft Bone in Sheep Spinal Anterior Interbody Fusion. *Spine (Phila Pa 1976)* 2002;27(6):567-575
2002	Toth	Evaluation of 70/30 poly (L-lactide-co-D,L-lactide) for use as a resorbable interbody fusion cage. *J Neurosurg Spine* 2002;97:423-432
2003	Assad	Porous titanium-nickel for intervertebral fusion in a sheep model- parts 1 and 2. *J Biomed Mater Res B Appl Biomater.* 2003 Feb 15;64(2):107-20
2005	Hojo	A biomechanical and histological evaluation of a bioresorbable lumbar interbody fusion cage. *Biomaterials* 2005;26:2643-2651
2005	Takahata	An Investigational Study on the Healing Process of Anterior Spinal Arthrodesis Using a Bioactive Ceramic Spacer and the Change in Load-Sharing of Spinal Instrumentation. *Spine (Phila Pa 1976)* 2005;30(8):E195-E203
2006	Lazennec	Evaluation of the 96/4 PLDLLA polymer resorbable lumbar interbody cage in a long term animal model. *Eur Spine J*. 2006;15:1545-1553
2006	Toth	Polyetheretherketone as a biomaterial for spinal applications. *Biomaterials* 2006;27:324-334
2007	Ito	Evaluation of hydroxyapatite ceramic vertebral spacers with different porosities and their binding capability to the vertebral body- an experimental study in sheep. *J Neurosurgery Spine* 2007;6(5):431-437
2008	Likibi	Influence of orthopaedic Implant Structure on Adjacent Bone Density and on Stability. *Am J Orthop* 2008;37(4):E78-E83
2008	Manunta	Lumbar interbody expanding cage - A preliminary study on an animal model. *Vet Comp Orthop Traumatol* 2008;21:382–384
2008	Strohm	Detection of Bone Graft Failure in Lumbar Spondylodesis - Spatial Resolution with High-Resolution Peripheral Quantitative CT. *AJR Am J Roentgenol* 2008;190:1255–1259
2008	Cunningham	Ceramic granules enhanced with B2A peptide for lumbar interbody spine fusion- an experimental study using an instrumented model in sheep. *J Neurosurg Spine* 2009;10:300-307
2009	Qian	Natural Bone Collagen Scaffold Combined with Autologous Enriched Bone Marrow Cells for Induction of Osteogenesis in an Ovine Spinal Fusion Model. *Tissue Engineering* 2009; 15(11);3547-3558
2010	Sherman	Evaluation of ABM/P-15 versus autogenous bone in an ovine lumbar interbody fusion model. *Eur Spine J* 2010;19:2156-2163
2011	Gu	Evaluation of an injectable silk fibroin enhanced calcium phosphate cement loaded with human recombinant bone morphogenetic protein-2 in ovine lumbar interbody fusion. *J Biomed Mat Res* 2011; 97A(2):177-185
2012	Solchaga	Augment Bone Graft Products Compare Favorably With Autologous Bone Graft in an Ovine Model of Lumbar Interbody Spine Fusion. *Spine (Phila Pa 1976)* 2012;37(8);E461-E467
2013	Fredericks	Assessment of bIoplex interbody Fusion devIce in a sheep lumbar fusion model. *Iowa Orthop J* 2013;33:33-39
2013	Humadi	A Comparison of Radiostereometric Analysis and calculated Tomography for the Assessment of Lumbar Spinal Fusion in a Sheep Model. *Evid Based Spine Care J* 2013;4:78–89
2014	Hong	New Cage for Posterior Minimally Invasive Lumbar Interbody Fusion- a Study in Vitro and in Vivo. *Orthop Surg* 2014;6:47–53
2015	Chen	Lumbar interbody fusion with porous biphasic calcium phosphate enhanced by recombinant bone morphogenetic protein-2/silk fibroin sustained-released microsphere- an experimental study on sheep model. *J Mater Sci Mater Med* 2015;26:126-138
2015	Yamada	A preclinical large animal study on a novel intervertebral fusion cage covered with high porosity titanium sheets with a triple pore structure used for spinal fusion. *Eur Spine J* 2015;24:2530-2537
2016	Bae	Transient Local Bone Remodeling Effects of rhBMP-2 in an Ovine Interbody Spine Fusion Model. *J Bone Joint Surg Am* 2016;98-A(24):2061-2070
2016	Wheeler	Allogenic mesenchymal precursor cells (MPCs) combined with an osteoconductive scaffold to promote lumbar interbody spine fusion in an ovine model. *Spine J* 2016; 16:389–399
2017	McGilvray	Evaluation of a polyetheretherketone (PEEK) titanium composite interbody spacer in an ovine lumbar interbody fusion model- biomechanical, microcomputed tomographical, and histological analyses. *Spine J* 2017; 17:1907–1916
2017	Pan	Cyst like osteolytic rhBMP-2 augmented sheep spinal fusion. *Am J Pathol* 2017, 187: 1485-1495
2018	McGilvray	Bony ingrowth potential of 3D-printed porous titanium alloy- a direct comparison of interbody cage materials in an in vivo ovine lumbar fusion model. *Spine J* 2018;18(7):1250–1260
2019	Aihara	Combustion Synthesis Porous Nitinol for Biomedical Applications. *Int J Biomaterials* 2019;1-11
2019	Gunzburg	Does nanoscale porous titanium coating increase lumbar spinal stiffness of an interbody fusion cage? An in vivo biomechanical analysis in an ovine model. *Clin Biomechanics* 2019;67:187-196
2019	Walsh	Does implantation site influence bone ingrowth into 3D-printed porous implants? *Spine J* 2019;19:1885-1898
2020	Grgurevic	Autologous blood coagulum containing rhBMP6 induces new bone T formation to promote anterior lumbar interbody fusion (ALIF) and posterolateral lumbar fusion (PLF) of spine in sheep. *Bone* 2020;138:115448
2020	Walsh	Undercut macrostructure topography on and within an interbody cage improves biomechanical stability and interbody fusion. *Spine J* 2020;20:18,761886
2021	Gadomski	Evaluation of lumbar spinal fusion utilizing recombinant human platelet derived growth factor-B chain. *JOR Spine*. 2021;4:e1166
2021	Kiapour	Bone Mineralization and Spinal Fusion Evaluation of a Truss-based Interbody Fusion Device. *Spine (Phila Pa 1976)* 2021;47(7):E319-E327
2021	Van Horn	Comparison of 3D-printed titanium-alloy, standard titanium-alloy, and PEEK interbody spacers in an ovine model. *Spine J* 2021;21:2097-2103
2022	Fogel	Subsidence and fusion performance of a 3D-printed porous interbody cage with stress-optimized body lattice and microporous endplates - a comprehensive mechanical and biological analysis. *Spine J* 2022;22:1028-1037
2022	Laratta	3D-printed titanium cages without bone graft outperform PEEK cages with autograft in an animal model. *Spine J* 2022;22:1016-1027
2022	Loenen	Early bone ingrowth and segmental stability of a trussed titanium cage versus a polyether ether ketone cage in an ovine lumbar interbody fusion model. *Spine J* 2022;22:174-182
2022	Loenen	Local bone metabolism during the consolidation process of spinal interbody fusion. *J Bone Min Metabolism* 2022;40:220-228
2022	Loenen	Peptide Enhanced Bone Graft Substitute Presents Improved Short-Term Increase in Bone Volume and Construct Stiffness Compared to Iliac Crest Autologous Bone in an Ovine Lumbar Interbody Fusion Model. *Global Spine J* 2022;12(7):1330-1337
2023	Matsugaki	Innovative design of bone quality-targeted intervertebral spacer- accelerated functional fusion guiding oriented collagen and apatite microstructure without autologous bone graft. *Spine J* 2023;23:609-620
2024	Christou	In vivo Assessment of AMP2, a Novel Ceramic-Binding BMP-2, in Ovine Lumbar Interbody Fusion. *Spine (Phila Pa 1976)* 2024;49(19):1381-1390

### Common study attributes

The main study design attributes are set out in Table V and in Supplementary Table i, with animal ages, interbody cages, fusion outcomes, and risk of bias discussed in more detail below.

**Table V.** Summary of key data extracted from the 48 studies examined in the review.

Variable	Most common	Second most common	Third most common
Breed	Merino (15%)	Suffolk (15%)	Dorset (4%)
Sex	Female (63%)	Male (8%)	
Levels fused	2 level (52%)	1 level (35%)	3 level (8%)
Assessment timepoints	6 months (54%)	3 months (44%)	4 months (44%)
Graft materials	Autograft (85%)	Ceramics (31%)	BMPs (-2, -6, -7) (23%)
Outcome measures	Radiological (96%)	Histological (92%)	Manual palpation and/or range of motion (52%)

### Animal ages

**Fig. 2:**
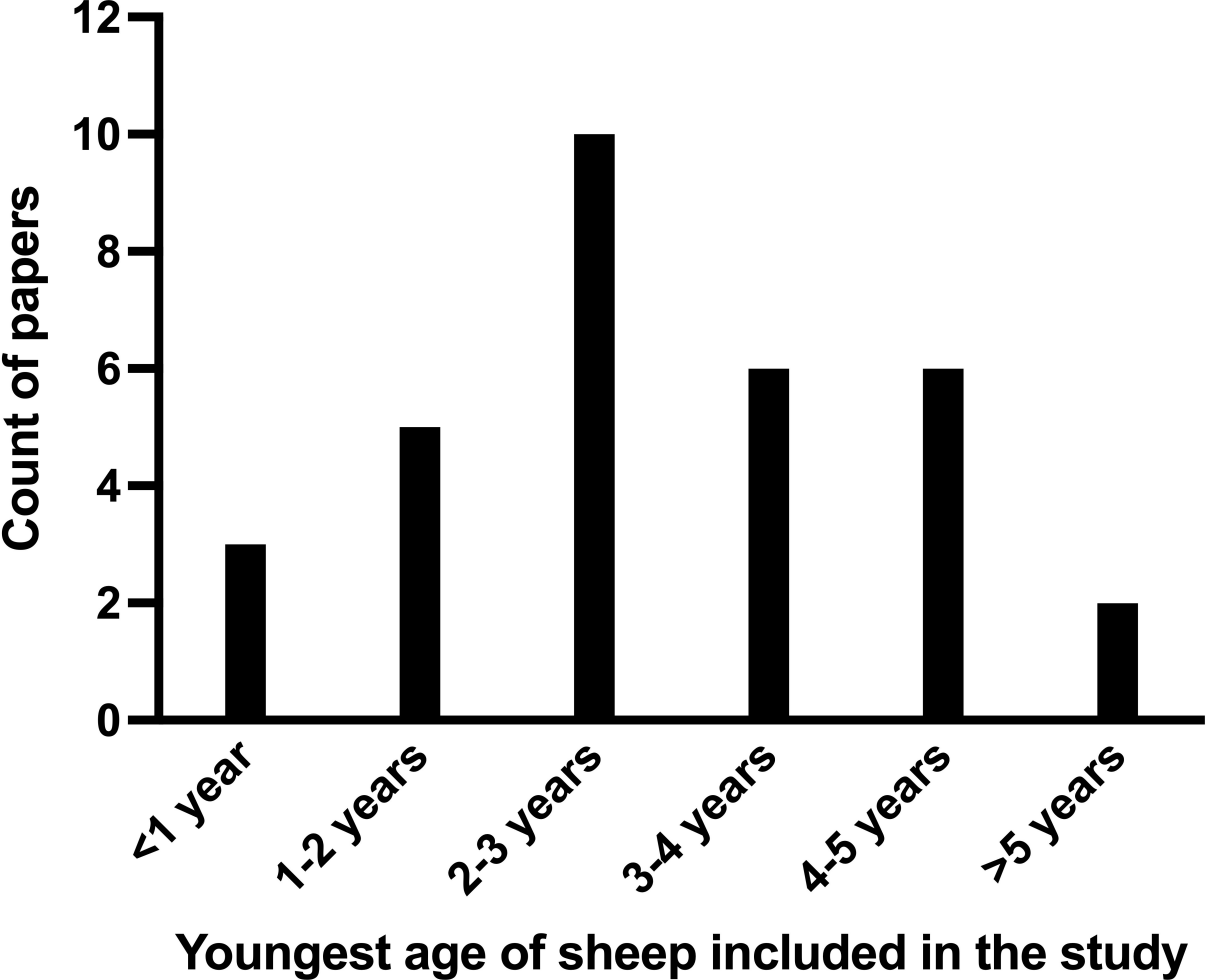
Analysis of the ages of sheep used in the studies covered by this review, split into groups based on the youngest age of each paper’s cohort.

Animal ages varied widely across studies, from six months to nine years; 16 studies did not report sheep ages, variably describing animals as “mature” or “skeletally mature”, or giving no age information at all, as was the case in five papers. The 32 studies that did report ages were grouped by the youngest animals in each and categorized, as shown in Figure 2. In eight studies, the within-study age range spanned two years or more.

### Interbody devices used

**Fig. 3:**
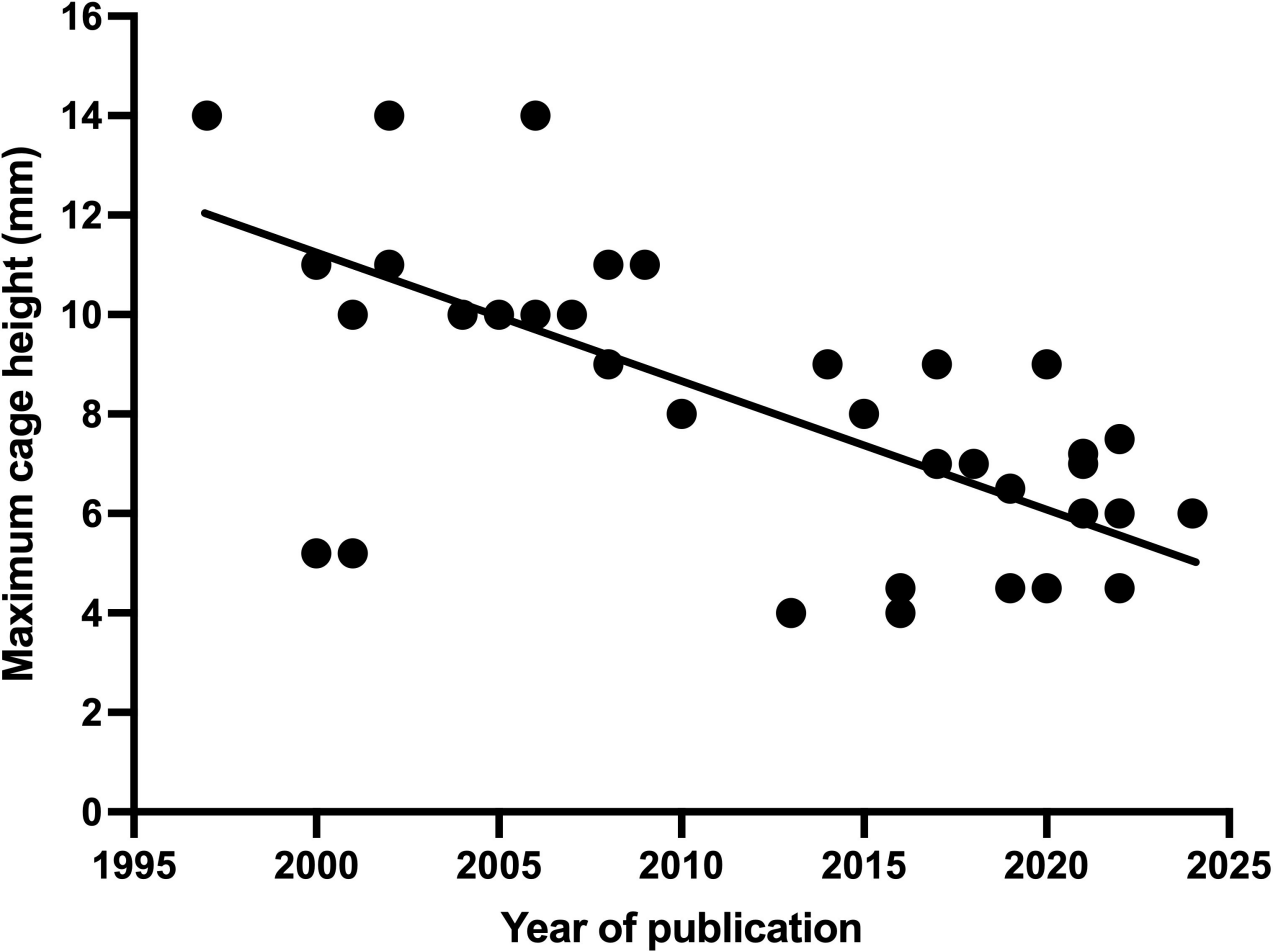
Analysis of the maximum cage height implanted per publication per year showing trend to lower cage height over time.

A variety of materials were used for interbody fusion devices, including polyetheretherketone (PEEK), titanium (Ti) alloys (TiAlV), 3D-printed porous Ti, Ti-coated PEEK, polymers, ceramic blocks, and porous nitinol. Cage dimensions also varied widely. In 15 studies, either no cages were used or dimensions were not reported. In the remaining 33, cage heights ranged from 4 mm to 14 mm. Threaded cylindrical cages, common in the 1990s and early 2000s, explain the larger sizes used early on. As PEEK gained popularity post-2000, cage heights decreased, reflecting the downward trend shown in Figure 3.

### Spinal fusion outcome measures

Radiological assessments included radiograph, conventional CT, and micro-CT (μCT). Mechanical evaluations involved manual palpation and range of motion (ROM) testing, most commonly applying 6 Nm (17% of studies), with forces ranging from 0.5 Nm to 10 Nm.

Device subsidence into vertebral endplates was observed in 37 of 48 studies (77%), with depths ranging from 1 to 2 mm to nearly complete collapse (up to 6 to 7 mm into both cranial and caudal vertebrae). Subsidence was most frequent in studies using autograft (95% of 42 studies) and slightly less so with recombinant BMPs (82% of 11 studies).

### Risk of bias assessment

The SYRCLE risk of bias (ROB) tool includes ten items covering selection, performance, detection, attrition, reporting, and other biases. Two authors independently assessed all 48 studies. Items lacking sufficient disclosure were scored as having potential bias (1), with total scores ranging from 0 to 10, where higher scores indicate greater risk. Discrepancies were resolved through discussion.

The aggregate average ROB score was 6.5 (SD 1.6) (Table VI), with frequent risks identified in allocation sequence, housing randomization, caregiver blinding, and outcome assessment randomization.

**Table VI.** Results of analysis of risk of bias using the SYRCLE risk of bias tool.

Item	Description of domain	Average score across all 48 papers (1 = risk of bias, 0 = no risk of bias)
1	Describe the methods used, if any, to generate the allocation sequence in sufficient detail to allow an assessment whether it should produce comparable groups.	0.69
2	Describe all the possible prognostic factors or animal characteristics, if any, that are compared in order to judge whether or not intervention and control groups were similar at the start of the experiment.	0.46
3	Describe the method used to conceal the allocation sequence in sufficient detail to determine whether intervention allocations could have been foreseen before or during enrolment.	0.96
4	Describe all measures used, if any, to house the animals randomly within the animal room.	0.98
5	Describe all measures used, if any, to blind trial caregivers and researchers from knowing which intervention each animal received. Provide any information relating to whether the intended blinding was effective.	0.96
6	Describe whether or not animals were selected at random for outcome assessment, and which methods to select the animals, if any, were used.	0.96
7	Describe all measures used, if any, to blind outcome assessors from knowing which intervention each animal received. Provide any information relating to whether the intended blinding was effective.	0.63
8	Describe the completeness of outcome data for each main outcome, including attrition and exclusions from the analysis. State whether attrition and exclusions were reported, the numbers in each intervention group (compared with total randomized animals), reasons for attrition or exclusions, and any re-inclusions in analyses for the review.	0.17
9	State how selective outcome reporting was examined and what was found.	0.08
10	State any important concerns about bias not covered by other domains in the tool.	0.67

**Fig. 4:**
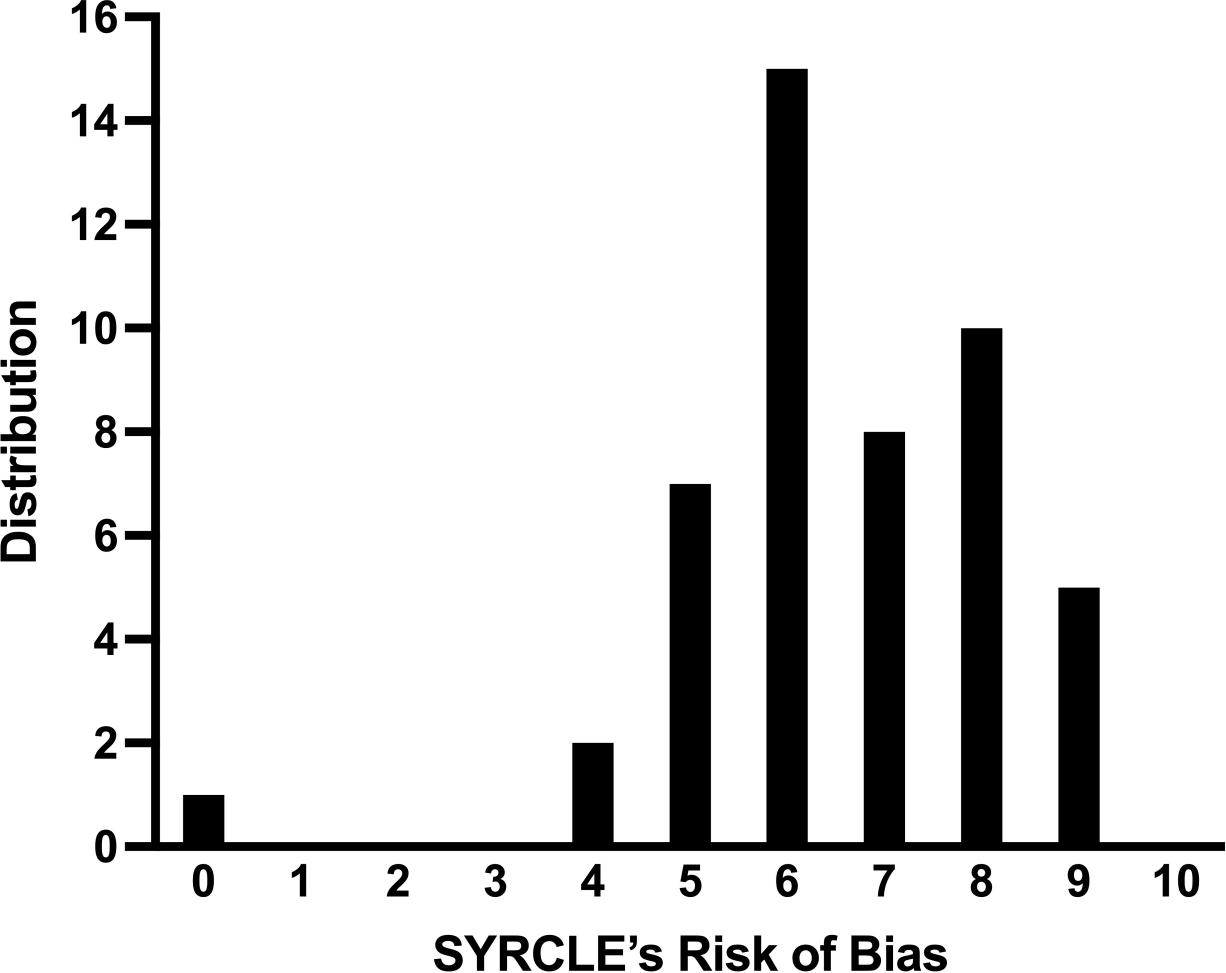
Distribution of the 48 papers in this review, analyzed using SYRCLE’s Risk of Bias tool. A score of 0 represents all questions adequately addressed, and a score of 10 indicates that none of the risk of bias questions were adequately addressed in the paper.

The distribution of papers by the degree of ROB is shown in Figure 4. A score of 0 indicates that paper adequately address all the SYRCLE’s questions, and a score of 10 indicates that the paper did not adequately address the ROB for any of the questions.

## Discussion

This review summarizes current practices in ovine LIF models, highlighting two critical factors – sheep skeletal maturity and cage size – that influence the clinical relevance of outcomes. It also addresses additional design considerations, including fusion assessments and risk of bias, highlighting factors which can increase transparency and comparability between studies.

### Sheep age

Preclinical models should replicate the human clinical scenario as closely as possible. Skeletal maturity in humans is often marked by third molar eruption (approximately 17 years).^18^ However, Bae et al,^19^ in a review of 196,118 patient records, found that the most common age group for spinal stenosis surgery was 65 to 84 years, with patients aged over 65 years accounting for 63.3% of procedures. Bone parameters change significantly with age, affecting density, structure, and strength.^20-22^ Fractures in juvenile animals with open growth plates heal more rapidly than in older animals, with regenerative potential declining sharply into adulthood.^23,24^ In sheep, Maenz et al^25^ reported significantly better bone volume, cortical thickness, osteoid volume and reduced osteoclast surface area in two- to four-year-olds compared to sheep aged over six years. Thus, selecting appropriately aged and skeletally mature sheep is essential to replicate clinical conditions in spinal fusion studies.

Ribitsch et al^11^ reported full skeletal maturity in sheep at three to four years. Koch et al^26^ found that 75% of two-year-old Merinos still had open growth plates, a confounding factor in orthopaedic studies. This appears to contradict limb-focused studies showing distal femur and proximal tibia growth closure at 18 to 26 months.^27^ However, Hammond and Appleton,^28^ as far back as 1932, noted that limbs mature before axial structures like the spine, explaining younger sheep’s “leggy” appearance. This discrepancy likely underlies incorrect assumptions about spinal maturity based on limb data. Supporting Ribitsch and Koch, Christou et al^29^ found that in sheep aged ≥ two years, vertebral growth plates were still open eight to 26 weeks after surgery. Accurate age reporting and assessment of skeletal maturity are therefore critical for proper interpretation of preclinical data.

This review found inadequate reporting of sheep age in interbody fusion models: 31% of studies omitted it. There were inconsistencies in assessing skeletal maturity, with 55% of studies using animals aged under three years that were therefore likely not skeletally mature in the spine, potentially overstating healing responses. Only 14% of studies clearly used spinally mature sheep.

### Interbody cage size and subsidence

Interbody cage subsidence is a serious complication of spinal fusions,^30^ often preventable by selecting an appropriately sized cage and preserving the vertebral endplates during disc preparation.^31^ To enhance the predictive value of ovine models, endplate preservation should be a key surgical goal. This requires understanding sheep spinal anatomy and selecting a cage no taller than the native disc height to avoid endplate breach and subsidence into vertebral cancellous bone.

**Fig. 5:**
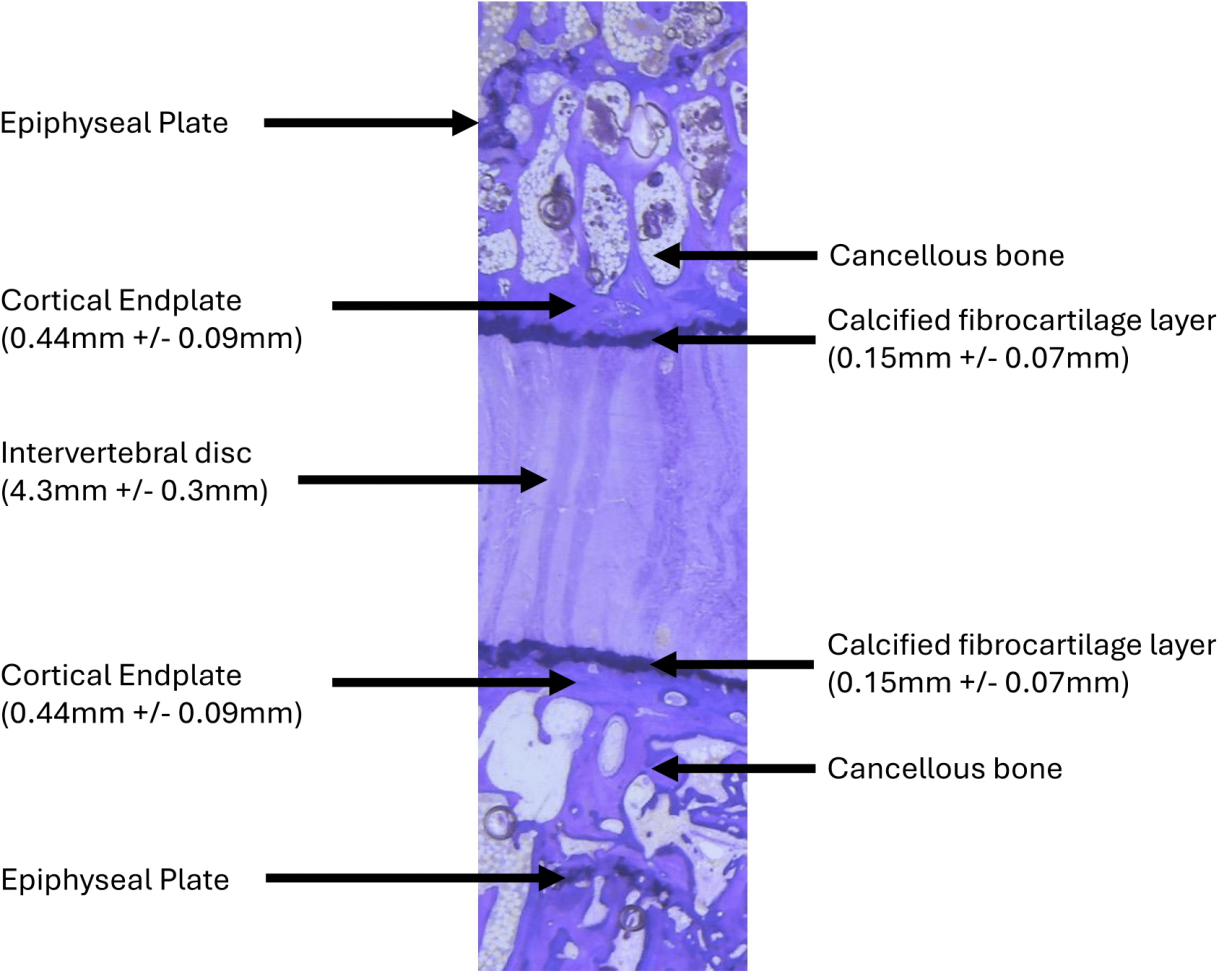
Low-magnification cross-section stained with methylene blue and basic fuchsin through a sheep L5/6 disc (μCT), with the average dimension for each layer cited from the literature.

A cross-section of a sheep spinal unit (L5/6) is shown in Figure 5. The vertebral bodies, principally consisting of cancellous bone, are separated from the nucleus and annulus by a thin endplate. The endplate consists of two distinct layers: the cortical bone of the subchondral endplate with a mean thickness of 0.44 mm (SD 0.09),^32^ and a calcified fibrocartilage layer with a thickness of 0.15 mm (SD 0.07).^33^

Wilke et al^15^ reported the anterior disc height of sheep. There is some variation by spinal level, ranging from 4.2 to 4.5 mm with the level most often chosen for fusion in sheep (L4/5) having a mean disc height of 4.3 mm (SD 0.3), and the second most commonly fused level (L2/3) having a disc height of 4.2 mm (SD 0.4).

**Fig. 6:**
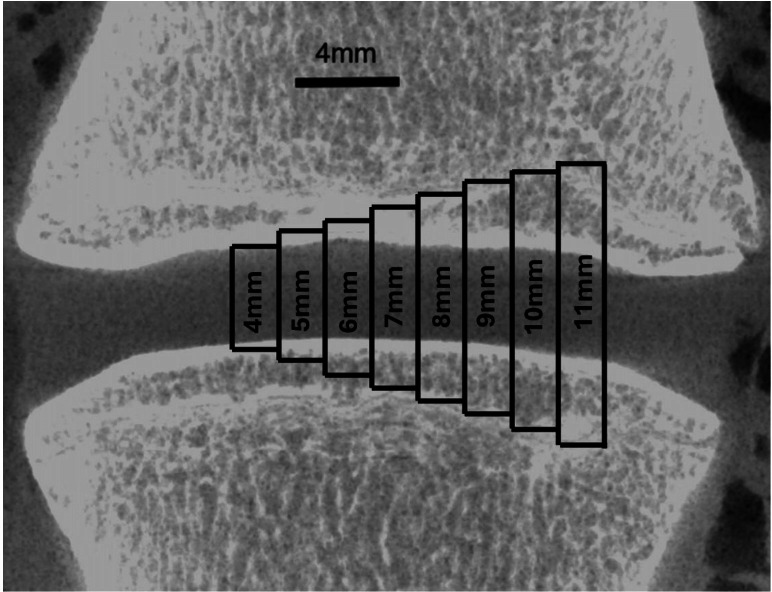
Representation of potential penetration through the endplates, at the L4/5 disc in a sheep, by cages > 5 mm in height.

However, the majority of cage heights identified in this literature review are larger than the height of the intervertebral discs. As Figure 6 shows, implantation of such oversized cages has the potential to disrupt the cortical subchondral bone of the endplates, especially cages ≥ 7 mm in height.

Several authors agree that endplate-sparing techniques more accurately replicate human clinical practice than the use of oversized cages.^29,34^ Oversized implants reduce translatability by altering the healing environment. Cages inserted into cancellous bone show different healing dynamics compared to those placed flush with the endplate cortex.^34^ Thus, heights over 5 mm can be anticipated to enhance bone formation,^34,35^ partly due to progressive subsidence. Assad et al^36^ and Hong et al^37^ reported average subsidence of 15.5% and 23.6%, respectively, over six months. In this review, 77% of studies with suitable images showed evidence of subsidence, likely due to the use of overly tall cages. This reduction in the interbody defect size may accelerate apparent healing by reducing the remaining intervertebral volume.

Sheep interbody cages typically lack integrated fixation screws, making them prone to instability and micromotion. Oversized cages may offer greater initial stability by engaging the cortical bone, thereby limiting micromotion, which is known to enhance osseointegration.^38^ However, their additional height also reduces leverage during ROM testing, potentially exaggerating mechanical performance compared to lower-profile, endplate-sparing cages.

### Endplate preparation

Successful graft incorporation depends heavily on adequate removal of the cartilaginous endplate (CEP). However, the CEP is thin (150.89 μm ± 69.25 in the lumbar region)^33^ and difficult to visualize intraoperatively, making surgical preparation challenging. Incomplete removal may compromise fusion, regardless of the cage or graft used.

The CEP tissue is avascular and unable to be actively remodelled.^39-42^ In a porcine model, Sinclair et al^43^ found that six of seven animals with no autograft remodelling had retained residual CEP within the cage. In sheep, the same group concluded that residual CEP on the endplate likely impedes bony integration of interbody devices.^33^

As noted by Steffen et al,^44^ the ovine LIF model is technically demanding and has a steep learning curve. Researchers must carefully remove the CEP while preserving the subchondral bone and avoiding penetration into cancellous bone. Postmortem histological analysis should be used to assess residual CEP and correlate its presence with fusion outcomes.

### Cage fit in an endplate-sparing model

Steffen et al^45^ reported that over half of specimens showed a fibrocartilaginous layer traversing much of the cage interior, often spanning its full cross-section. Unlike human surgery, where trial cages help to ensure proper sizing, most sheep studies use a single cage size. Given inter- and intra-sheep disc height variability (Figure 6), this often results in poor cage-to-endplate fit, promoting micromotion or graft displacement, leading to incomplete fusion caused by fibrous tissue ingrowth.^45^

Poor endplate contact may also contribute to lower radiological fusion scores in endplate-sparing models, thus emphasizing the need for careful histological assessment.

**Fig. 7:**
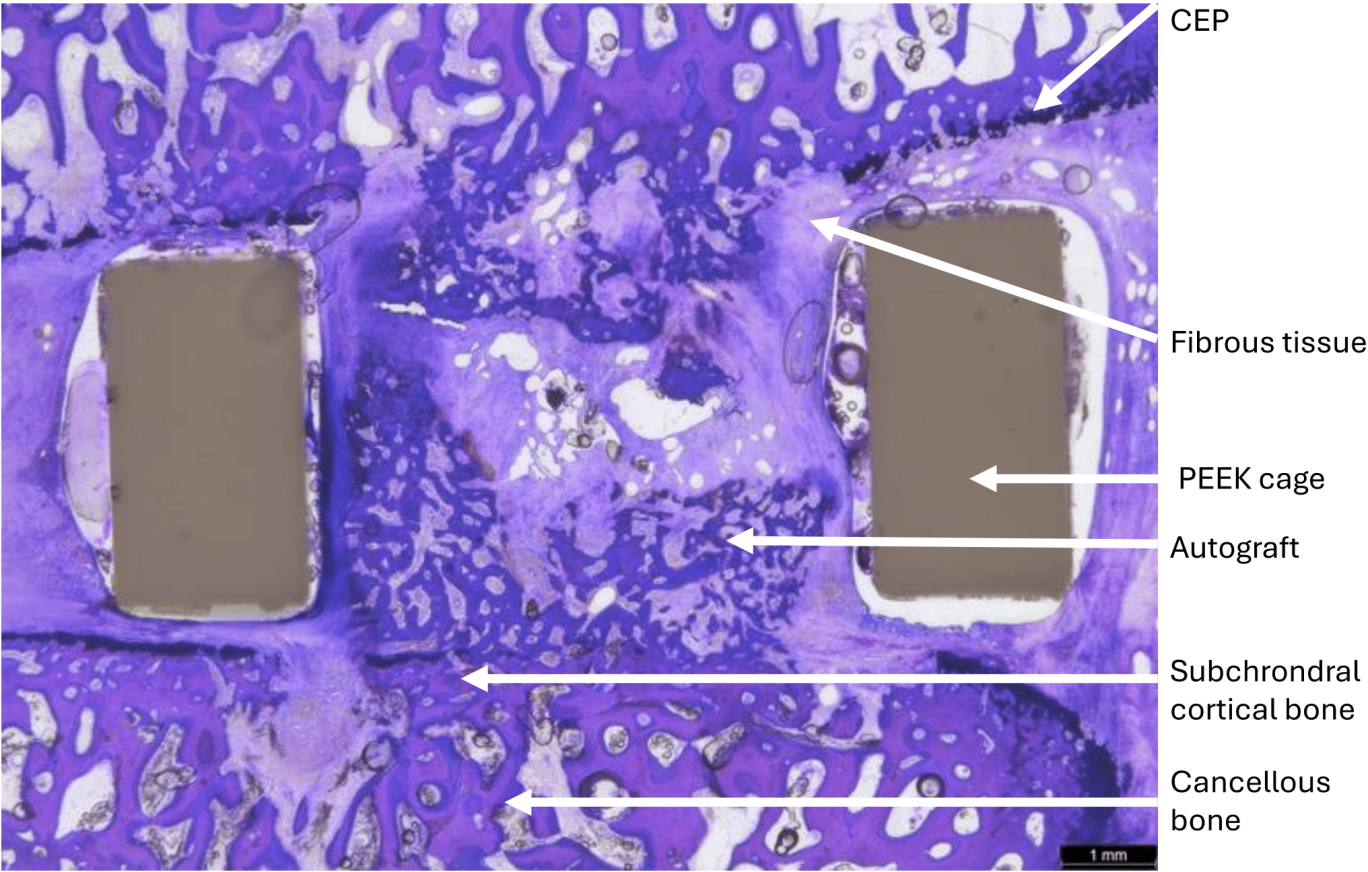
Histological assessment of an interbody fusion cage in a sheep model with inadequate endplate fit, leading to autograft resorption and arthroplasty with fibrous tissue. CEP, cartilaginous endplate; PEEK, polyetheretherketone.

Figure 7 shows a histological section of a PEEK cage filled with autograft at two months. While the CEP appears adequately removed, the wedge-shaped disc results in a poor fit on the right, where autograft resorption and fibrous tissue infiltration are evident.

### Interpreting ROM data

Hueng et al^46^ showed that interbody cage placement, be that anterolateral, mediolateral, or posteromedial, can affect ROM outcomes in large animal models. Researchers should consider analyzing ROM results in relation to cage position to identify and account for potential outliers.

### Fusion assessment: radiography vs histology

Beyond the key design factors of animal age and cage size, the method of assessing fusion is also critical. The reviewed studies employed various techniques, including radiograph, conventional CT, μCT, and histology. Studies using multiple methods allowed within-animal comparisons, revealing that conventional CT tends to overestimate fusion relative to μCT,^29,47^ making μCT the preferred method for future studies.

Humadi et al^48^ reported substantial inter- and intraobserver variability in CT-based fusion assessments, and a 37% disagreement between fine-cut CT (0.25 mm slices) and histology. CT overestimated fusion 33% of the time, while histology only identified fusion when CT did not in 4% of cases, underscoring the importance of histological validation.

Despite μCT’s advantages, fusion scoring scales vary widely, ranging from binary to nine-point scales, limiting cross-study comparability. The most frequently used scale (36%) was a four-point system (Table VI). Standardizing μCT scoring, as shown in Table VII along with reporting voxel resolution, would reduce partial volume effect errors and improve consistency across studies.

**Table VII.** Four-point scale applied to µCT results to assess degree of fusion through an interbody fusion cage.

Score	Grade	Description
0	No new bone	No new bone formation visible
1	Visible new bone	New bone formation visible, but no continuous bone
2	Possible fusion	Continuous bilateral bridging new bone formation with visible lucency
3	Probable fusion	Continuous bilateral bridging new bone formation

Standardization of key endpoints, such as three- and six-month timepoints and use of 6 Nm in ROM testing, would further improve the comparability and interpretability of preclinical spinal fusion research.

### Translatability or utility score

To support both readers and future researchers, we propose a framework for rapidly assessing the translational utility of preclinical studies (Table VIII). This review highlights two key controllable factors that can significantly affect study outcomes: skeletal maturity of the sheep, and interbody cage size. We therefore assigned a greater weighting to these two variables as set out below. We also consider the use of the gold standard of autograft as a control and adequate fusion assessments to be of value to readers of these preclinical studies.

**Table VIII.** Nottingham Utility Score to be used to assess the translatability of a sheep lumbar interbody fusion study to human clinical practice.

Element	Description	Score
Sheep age	Sheep age not reported or < 3 years old	-2
	Sheep 3 to 4 years old	0
	Sheep > 4 years old	+2
Cage height	Cage height not reported or > 5 mm	-2
	< 5 mm	+2
Fusion assessment	Only μCT or mechanical testing	-1
	Both μCT and mechanical testing	+1
Autograft control	Iliac crest bone graft included	+1

**Fig. 8:**
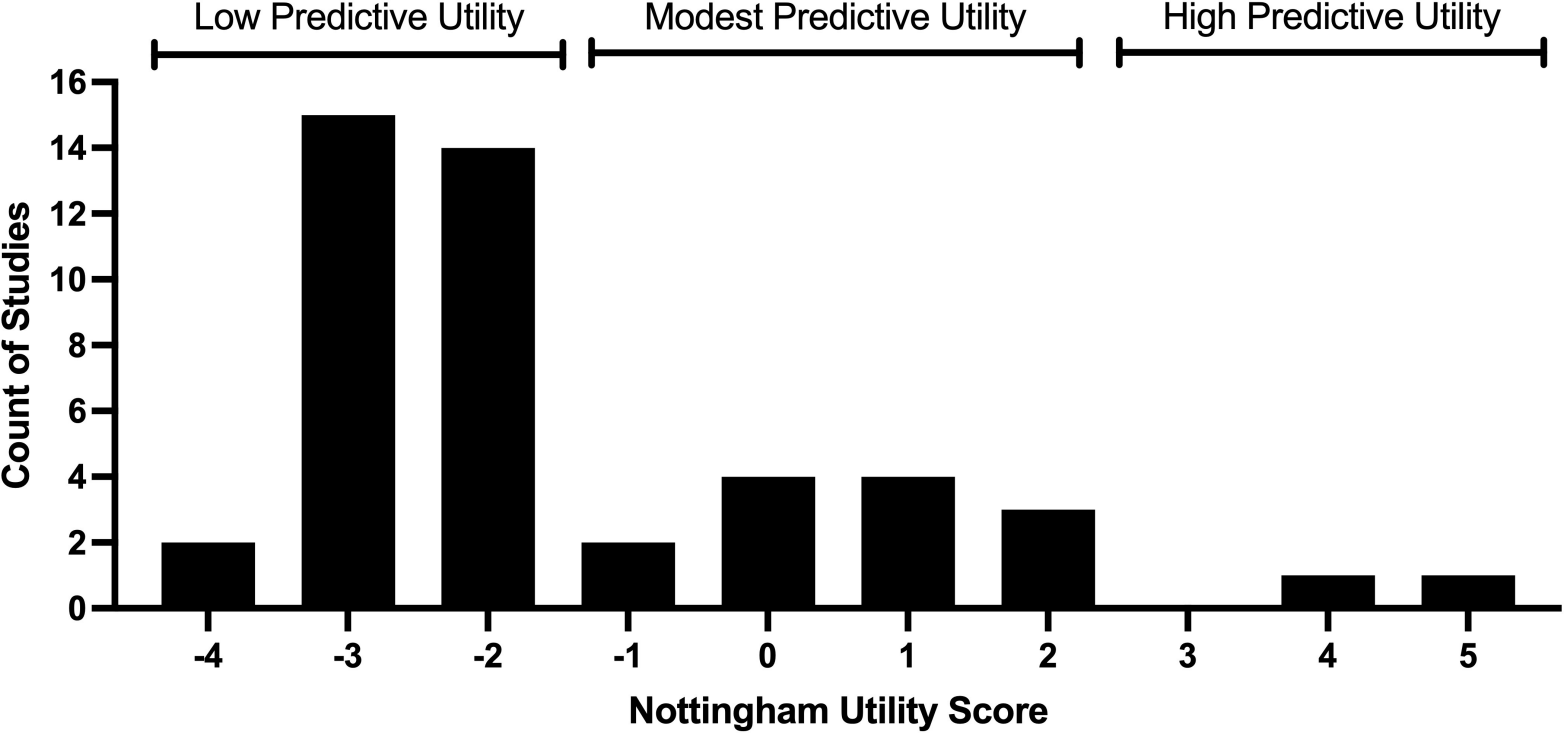
Distribution of papers analyzed using the Nottingham Utility Score as an indicator of predictive utility for translation of the preclinical results based on similarity with the clinical model.

Applying this scoring method, results from the 48 studies reviewed are presented in Figure 8.

Due to frequent non-disclosure of these variables, and the common use of immature animals and oversized cages, many of the studies were rated as having limited translational value to human clinical practice. There has been no improvement in the scores over time (Supplementary Figure a).

### Risk of bias

In addition to the low utility of many of the studies that have been reviewed, there is also a high rate of significant ROB. Fewer than 50% of the papers demonstrated low or intermediate scores. ROB could be substantially improved with comprehensive disclosures of the methods employed to reduce bias and thus increase confidence in the papers. Despite the publication of the Animal Research: Reporting of in vivo Experiments (ARRIVE)^49^ guidelines in 2010, and the subsequent publication of SYRCLE’s risk of bias tool for animal studies in 2014,^17^ there has been no general improvement over time in the calculated ROB scores (Supplementary Figure b).

### Translational gaps and clinical relevance

While ovine LIF models replicate the fundamental surgical approach of human LIF procedures—disc removal and arthroplasty with interbody spacers and bone graft—this paper shows that important gaps remain between current preclinical models and clinical practice. Specifically, the typical human LIF patient is elderly (65+ years)^19^ with degenerative disc disease and increased comorbidity burden.^50^ In contrast, 55% of reviewed studies used skeletally immature sheep (< 3 years), creating a ‘best-case scenario’ that may overestimate fusion efficacy relative to the target clinical population.^23-25^

Current models also often fail to replicate human surgical precision. Human LIF procedures employ careful endplate preparation with cage sizing matched to native disc height, whereas the majority of sheep studies violated the endplates with oversized cages and showed evidence of subsidence. This oversizing artificially enhances mechanical stability and accelerates apparent fusion through gap closure, potentially masking the true performance of test materials.

These translational gaps have important implications for clinical translation. Enhanced healing in young animals combined with oversized cage effects may lead to overoptimistic efficacy predictions, potentially contributing to clinical trial failures or suboptimal patient outcomes.

Ovine LIF models are widely used in developing implants and bone graft substitutes for spinal fusion. However, variability in animal age, surgical technique, cage size, fusion assessment methods, control use, and study duration make comparisons difficult. Many studies are biased toward higher fusion rates due to oversized cages in immature animals, potentially overstating efficacy and posing risks in early human trials.

The ARRIVE guidelines from the NC3Rs offer a foundation for robust experimental design and reporting.^49^ Building on this, our review identifies key considerations specific to ovine spinal fusion models. We recommend the following: animal age > four years; consider using two non-consecutive level fusions, aligned with the 3Rs (replace, reduce, refinement); disclosure of cage size and use of the endplate sparing technique that mimics clinical practice; employ two methods of assessing fusion (histology and μCT); standardize ROM testing force at 6 Nm; standardize the radiological scoring method for μCT; include a positive control iliac crest bone graft; standardize the fusion assessment endpoints at three and six months; improve disclosure of bias mitigation methods according to SYRCLE’s ten elements; and self-reporting of the paper’s score against the SYRCLE’s RoB assessment tool.

## Data Availability

The data that support the findings for this study are available to other researchers from the corresponding author upon reasonable request.
